# Preliminary findings of a randomized controlled trial investigating the efficacy of transcranial magnetic stimulation in treatment-resistant depression: a *post-hoc* analysis on the role of co-occurring personality disorders

**DOI:** 10.3389/fpsyt.2024.1363984

**Published:** 2024-11-11

**Authors:** Julian Maciaszek, Joanna Rymaszewska, Tomasz Wieczorek, Patryk Piotrowski, Dorota Szcześniak, Jan A. Beszłej, Monika Małecka, Bogna Bogudzińska, Adrianna Senczyszyn, Damian Siwicki, Marta Biercewicz, Krzysztof Kowalski, Anna Zimny, Przemysław Podgórski, Karolina Fila-Pawłowska

**Affiliations:** ^1^ Department of Psychiatry, Wroclaw Medical University, Wrocław, Poland; ^2^ Department of Clinical Neuroscience, Faculty of Medicine, Wroclaw University of Science and Technology (WUST), Wrocław, Poland; ^3^ Department of Radiology, Wroclaw Medical University, Wroclaw, Poland

**Keywords:** transcranial magnetic stimulation, TMS, theta burst stimulation, TBS, major depressive disorder, treatment-resistant depression, personality disorders

## Abstract

**Introduction:**

Despite the high hopes for the use of transcranial magnetic stimulation (TMS) in the treatment of depression, between 30% and 60.5% of patients do not respond to stimulation. The factors contributing to non-response, especially those related to personality, remain insufficiently investigated. The main aim of our study was to compare the efficacy of active TMS and sham–placebo protocols in combined therapy of treatment-resistant depression with evaluation of possible personality disorders comorbidity.

**Methods:**

The study was conducted between December 2019 and December 2022, as a randomized, double-blind, active comparator-controlled and sham-controlled parallel trial. Patients (n = 41) were randomized into one of two experimental conditions (active TMS vs. placebo) and screened before and after stimulation as well as at a 3-month follow-up. Personality disorders were assessed with The Structured Clinical Interview for DSM-5 Personality Disorders.

**Results:**

There were no significant differences between the TMS active and sham groups in terms of general characteristics, coexisting personality disorders, and Montgomery–Åsberg Depression Rating Scale scores before stimulation, at the end of stimulation, and after 3 months of stimulation. However, linear regression analysis revealed significant negative associations between the coexistence of personality disorders and the reduction of depressive symptoms from baseline to the end of stimulation. The post-hoc exploratory analysis on the first phase of the RCT confirmed the presence of personality disorders to be a consistent negative influence on the reduction of depressive symptoms post-stimulation, regardless of protocol and experimental condition and demonstrated a smaller percentage reduction in depressive symptoms after stimulation in patients with personality disorders.

**Discussion:**

A central conclusion, based on our study, is that transcranial magnetic stimulation for treatment-resistant depression cannot be considered as a method independent of co-occurring personality disorders.

## 1 Introduction

Transcranial magnetic stimulation (TMS) is a widely accepted treatment method for major depressive disorder (MDD), with its efficacy confirmed by numerous controlled clinical trials (1). TMS is a technique of non-invasive brain stimulation. With the use of electromagnetic impulses generated by a special coil, penetrating the skin, skull bone, and meninges into the cerebral cortex, an electric current is induced in the neighboring nerve cells. The impulse affects the tissue directly at the site of application, and thanks to numerous connections with many other structures, it can spread into further regions and functional brain networks (2).

### 1.1 TMS protocols

The original U.S. Food and Drug Administration (FDA)-approved repetitive transcranial magnetic stimulation (rTMS) protocol for MDD, with a high frequency of 10–20 Hz applied over the dorsolateral prefrontal cortex (DLPFC), has proven to have significant practical limitations. The long stimulation duration (30 sessions of 37.5 min each) makes it difficult to apply in daily clinical practice (3–6). A more recent alternative, the theta burst stimulation (TBS) protocol also applied over the DLPFC, increases cortical excitability in a more robust and longer-lasting way than rTMS and in a similar way to high-frequency (HF) rTMS. It offers the prospect of a significant reduction of the stimulation time (i.e., to 40 sessions of 3 min each), due to the use of higher frequencies of pulse emission (7). Recent studies demonstrate TBS protocols to be safe, for up to 10 sessions per day (8). In August 2018, the US FDA approved intermittent theta burst stimulation (iTBS) for the treatment of adults with treatment-resistant depression (TRD) (9) and, in September 2022, cleared the SAINT Neuromodulation System for the treatment of refractory depression in adults (10). SAINT is an innovative form that combines MRI-guided selection of the targeted brain region with an accelerated stimulation regimen involving 10 short iTBS sessions every day for 5 days. Previous studies also suggest that iTBS was non-inferior to 10-Hz rTMS for the treatment of depression (11, 12).

### 1.2 Mechanisms of TMS action

There are several putative mechanisms of actions of rTMS described so far, mainly associated with the influence on neurotransmission, modulation, and neuroplasticity of several brain areas (13).

#### 1.2.1 Brain activation changes

Using brain imaging, Xue et al. (14) found that compared to sham rTMS, high-frequency rTMS of the left DLPFC increased the activation of the anterior cingulate. Similarly, Bridges et al. (15) found that left DLPFC rTMS increases the activation levels of sensory and motor cortical areas in healthy participants. Several studies in healthy individuals found that rTMS to the left DLPFC increases cognitive control (16), possibly due to an increase in frontal cortical activation.

#### 1.2.2 Dopamine level

In addition to modulating the activation of various brain areas, several studies have provided strong evidence that left DLPFC TMS modulates dopamine levels in cortical structures such as the anterior cingulate cortex (ACC), medial orbitofrontal cortex (17), and subcortical structures, e.g., the ventral tegmental area and the substantia nigra (18). The DLPFC participates in the reward and decision-making circuits that are associated with the integration of cognitively and motivationally relevant information and the inhibitory control over pursuing immediate reward (19, 20). Several studies found that rTMS increases dopamine levels within the mesolimbic system, anterior cingulate cortex, and caudate nucleus (17, 21–23). One recent review shows that rTMS increases dopamine levels in the basal ganglia (24).

#### 1.2.3 Connectivity

rTMS could modulate cortical–subcortical connectivity, especially within the putamen, thalamus, and cerebellum (25). Chen and colleagues examined the effect of rTMS on three cortical networks: fronto-parietal central executive network (CEN), cingulo-opercular salience network (SN), and the default mode network (DMN) and their interplay (26). The dorsolateral prefrontal node, which is situated within the CEN, was found to inhibit CEN interactions primarily with the DMN. The resting DMN activity was altered after inhibitory rTMS to a regulatory node in the CEN. Finally, the resting state relationship between the CEN/SN and DMN was found to be modifiable by rTMS, which confirms that DLPFC rTMS has impacts on the activation levels of subcortical areas. Furthermore, a reduction of hyperconnectivity between the DLPFC and the DMN was found in patients with depression who responded to DLPFC rTMS (27, 28). The DMN plays a key role in regulating rumination, self-referential processing, and episodic memory retrieval and includes the medial prefrontal cortex, posterior cingulate cortex, and mostly medial areas of the posterior parietal cortex (29).

In contrast, the CEN is responsible for regulating attention, working memory, and decision-making (30). DLPFC as part of CEN is usually recruited during the performance of tasks that require control over interference (31). Along these lines, Williams (32) described the cognitive control network to consist of DLPFC as well as the default mode and attention network components such as the ACC and the inferior parietal lobules. They also suggested that ACC plays a key role regarding the DLPFC iTBS treatment of the core depressive symptoms which is hypothesized to be mediated in part through indirect inhibitory functional connectivity from the left DLPFC to the subgenual anterior cingulate cortex (sgACC) (33, 34). Strong evidence explaining TMS action was provided by Tik et al. (35) who performed a study on healthy individuals. They concluded that rTMS over DLPFC causes transient changes to only one resting state node (RSN), namely, RSN#17 comprising the DLPFC and ACC. The ACC is interlinked to structures such as the meso-cortico-limbic dopamine system following rTMS stimulation. Similarly, an attenuation of initial hyperactivity of the subgenual cingulate and other cortical areas among MDD individuals was observed after TMS stimulation was correlated with depressive symptom reduction (36).

### 1.3 Efficacy of TMS

Despite high hopes for the use of magnetic stimulation in the treatment of MDD, according to research, between 30% and 60.5% of patients do not respond to stimulation (37). Therefore, apart from establishing an effective and practically applicable stimulation protocol, identifying the predictors of TMS efficacy appears to be an urgent topic for current clinical trials. Some methodological difficulties in assessing the efficacy of TMS protocols in MDD stem from the heterogeneous nature of depression. Available studies highlight the central role of the subgenual anterior cingulate cortex, default mode network, and salience network as predictors of TMS response and suggest their involvement in the mechanisms of antidepressant action (38). Drysdale et al. (39) demonstrated a varying efficacy of TMS protocols, depending on patient affiliation to one of four neurophysiological subtypes (“biotypes”) defined by a specific symptom profile along with distinct patterns of dysfunctional connectivity in the limbic and front striatal networks (40).

### 1.4 Personality disorders as a confounding factor

Another obstacle in assessing the available TMS MDD studies is the fact that most of the published studies, including reviews and meta-analyses, rely solely on the assessment of the severity of depressive symptoms and do not include an evaluation of coexisting personality disorders (26, 41–43). The presence of personality disorders (PDs) neither excludes the possibility of patients experiencing one or more depressive episodes nor decreases the likelihood of such comorbidities. On the contrary, PDs and MDD have been commonly occurring coexisting disorders among mental health disorders (44–46). According to former studies, up to three-fourths of patients with PDs have suffered from at least one depressive episode (47, 48). The more frequent occurrence of depressive episodes may result, depending on the type of PD, from a disturbed activity of neural networks especially related to dysfunction of the fronto-limbic circuitry and the default mode network as in borderline personality disorder (BPD) (49, 50) or other psychological variables that may make PD patients more vulnerable to depressive symptoms. In axis I disorders and MDD, coexisting PDs are associated with a number of additional factors, such as higher levels of general psychopathology, greater impairments in social and occupational functioning (51, 52), and results in a poorer treatment response across various treatment methods (53, 54). Still, studies assessing the impact of comorbid PDs on the efficacy of TMS in the treatment of depressive episodes are scarce. In one of the available studies, the authors found a group of patients with BPD who received TMS protocols of various durations without a placebo control (55). The authors concluded that comorbid BPD had no significant influence on the efficacy of TMS in the reduction of depressive symptoms. It is, however, worth noting that the BPD diagnosis was based on a retrospectively performed self-assessment questionnaire, without any clinical psychological evaluation. The limitations of the aforementioned study prompted us to conduct a similar study on a smaller scale but based on a randomized controlled trial (RCT) design including a clinical psychological diagnosis of co-occurring PDs and prestimulation. In another case study, improvements were observed in the antidepressant efficacy among patients with BPD (56). Feffer et al. in their small sample, crossover sham-controlled trial suggested efficacy for dorsomedial prefrontal cortex (DMPFC)-rTMS in treating MDD in BPD (57). In their limited-scale investigation (*n* = 15), Reisenberg et al. (58) indicated the efficacy of 1-Hz rTMS directed toward the orbitofrontal cortex (OFC) in addressing depressive symptoms and symptoms of BPD, encompassing both antidepressant treatment and impulsivity reduction. However, attention was drawn to an almost 50% dropout rate and poor stimulation tolerance. On the other hand, Bulteau et al. (59) in their investigation concluded that the influence of personality disorders and/or prior suicide attempts on the negative self-reference domain highlighted the significance, from the patient’s standpoint, of these two variables in the prognosis of MDD, irrespective of the biological treatment modality employed (such as 1 Hz of right-brain DLPFC stimulation and antidepressants). The causes for non-response to TMS, with particular emphasis on personality factors, to TMS continue to be inadequately understood. In light of the above, we discern notable gaps in the quantity of prior research addressing the role of personality disorders in the treatment of depression using TMS. In order to fill this gap in our study, we focused on the detailed psychological examination of patients regarding the coexistence of personality disorders.

The aim of our preliminary study was to compare the efficacy of active TMS and sham–placebo protocols in combined therapy of treatment-resistant depression with evaluation of possible personality disorders comorbidity.

## 2 Materials and methods

The study was conducted between December 2019 and December 2022 in a randomized, double-blind, active comparator-controlled and sham-controlled parallel trial design. Raters and participants were both blinded to the randomization procedure and therefore unaware of the type of received stimulation. Due to technical considerations, the person applying the stimulation was unblinded and not involved in any rating activities. The study protocol was registered with ClinicalTrials.gov Identifier: NCT05543421. Here, we present the *post-hoc* exploratory analysis of the first phase of the RCT.

### 2.1 Participants

The information about the study and the possibility of participation was made available in the form of paper brochures at the WMU Psychiatry Outpatient Clinic and other outpatient psychiatric clinics in Wroclaw as well as online, on the WMU’s Psychiatry Dept. website and the WMU’s and Polish Psychiatric Society’s social media accounts. Patients with a diagnosis of recurrent depression were asked to fill out an online application for the clinical trial providing their contact details and sociodemographic information. The patients who completed the application form were contacted and examined by a psychiatrist to verify the inclusion and exclusion criteria and to assess the severity of depression symptoms at T0 (before stimulation). The inclusion criteria include diagnosis of MDD according to the ICD-10 criteria; a total score of the Hamilton Depression Rating Scale (HAM-D) of at least 17 points (60); and treatment resistance—no remission in MDD symptoms despite 4–6 weeks of pharmacological therapy with selective serotonin reuptake inhibitors, serotonin norepinephrine reuptake inhibitors, or tricyclic antidepressants in adequate dosages and stabilized pharmacotherapy with no dosage changes for at least 1 month prior to recruitment. The patients also had to be between the ages of 18 and 70 years. The exclusion criteria were defined as follows: lack of informed consent from the patient and a documented persistent lack of adherence; history of stroke or head injury with neurological deficits; diagnosis of increased intracranial pressure or a positive history of increased intracranial pressure; current or lifetime diagnosis of any other serious mental disorder; actively suicidal in the judgment of the investigator; acute, serious, or unstable medical conditions; pregnancy or lactation period; positive history of seizures or a positive family history of epilepsy; magnetic or ferromagnetic implants, both electronic (e.g., heart/brain stimulators) and mechanical (e.g., bone anastomoses) within the head and neck in the stimulation range, and additional MRI contraindications [claustrophobia, heart pacemakers and other electronic devices implanted in the body, ferromagnetic metal foreign bodies in soft tissues (especially the eyeball), aneurysmal clips or coils within cerebral arteries, any metal implants within head and neck area including braces on teeth]. The patients who were included in the study also received a structural MRI in order to exclude radiological contraindications to stimulation as well as a functional magnetic resonance imaging (fMRI) for research purposes.

### 2.2 Protocols

In the next step, the patients were randomized into one of two groups with a given probability, using the Sealed Envelope (61) website: the TMS active group (66.7%) and the TMS sham group (33.3%), and randomization was additionally stratified by sex, age, and severity of depressive symptoms at baseline (T0). Patients from the active TMS group received 50% rTMS and 50% iTBS both over the DLPFC. Due to available reports indicating no significant differences in the efficacy of both protocols in the treatment of MDD (11, 12) and due to the preliminary character of our study and relatively small sample, we decided to collectively analyze patients with active stimulation, treating the stimulation type as an independent variable in our analysis.

rTMS stimulation using a PowerMAG TMS Therapy stimulator (MAG & More, Germany) equipped with a figure-of-eight coil was performed within the left hemisphere, over the DLPFC according to FDA recommendations (6) using the so-called “train stimulation” (stimulation of a given frequency for a short period of time with longer breaks without stimulation) with the following stimulation parameters: magnetic field intensity, 120% of the initial excitability threshold for the motor cortex; frequency, 10 Hz for 4 s of stimulation; breaks in stimulation, 26 s; total number of stimulation pulses, 3,000; duration of one session, 37.5 min; and duration of therapy, 6 weeks (sessions on days 1–5, break on days 6 and 7).

The stimulation with the accelerated iTBS protocol was performed within the left cerebral hemisphere, over the DLPFC with the following stimulation parameters: 50 Hz frequency; intensity, 80% excitability threshold for the motor cortex; breaks between pulse series, up to 10 s; duration of a single session, from 20 to 190 s; duration of treatment, 40 sessions; total number of pulses in a session, 600; sessions a day, 4; intersession interval scheme, 2 sessions with 10 min intersession interval, 2 h break and another 2 sessions with 10 min intersession interval; duration of one session, 3 min; and duration of therapy, 2 weeks (sessions on days 1–5, break on days 6 and 7).

In the control group, we used the sham TMS coils that resembled regular TMS coils but were equipped with a magnetic shield that attenuated the magnetic field (62). As a consequence, these sham TMS coils were positioned exactly like an active TMS coil resulting in a very good approximation of the auditory effects as well as somatosensory effects and peripheral nerve stimulation that occurred during the application of active TMS.

Before stimulation, the excitability threshold was determined within the motor cortex using electromyography. The target of stimulation, the DLPFC, was localized according to the “6-cm rule”: stimulating the motor cortex and recording motor evoked potentials in the contralateral hand muscle (i.e., the abductor pollicis brevis) and then measuring 6 cm anterior from this position along a parasagittal line (63, 64).

### 2.3 Measures

The patients underwent a psychiatric examination at three points in time: T0—before stimulation, T1—at the end of stimulation, and T2—3-month follow-up, after the end of treatment. The severity of depressive symptoms was assessed using The Montgomery–Åsberg Depression Rating Scale (MADRS). The MADRS is a 10-item diagnostic tool, which allows for a better insight into the patient’s mood, energy, and motivation levels (65). The patients were also subjected to psychological evaluation for the coexistence of personality disorders using The Structured Clinical Interview for DSM-5 Personality Disorders (SCID-5-PD) (66), which was conducted before stimulation. We decided that the assessment of patients by a trained psychologist was necessary because personality disorder should not be solely based on one test or questionnaire but most importantly through a clinical interview.

### 2.4 Statistical analyses

The Mann–Whitney *U* test (for continuous variables) and the chi-square or Fisher’s exact test (for qualitative variables) were used to compare the active and sham TMS groups. Due to multiple comparisons, the Bonferroni correction was applied to the level of significance. Taking into account six bivariate comparisons, the level of significance was finally set at 0.008. The analysis of covariance (ANCOVA) was performed to investigate the differences in the levels of depressive symptom reduction at the end of stimulation and after 3 months of follow-up, between patients receiving active and sham stimulation after co-varying for potential confounding factors. A multiple stepwise linear regression was performed to identify variables associated with the antidepressant effect of TMS. The results were considered significant if the *p*-value was less than 0.05. All analyses were performed in JASP.

## 3 Results

### 3.1 CONSORT diagram

**Figure 1:**
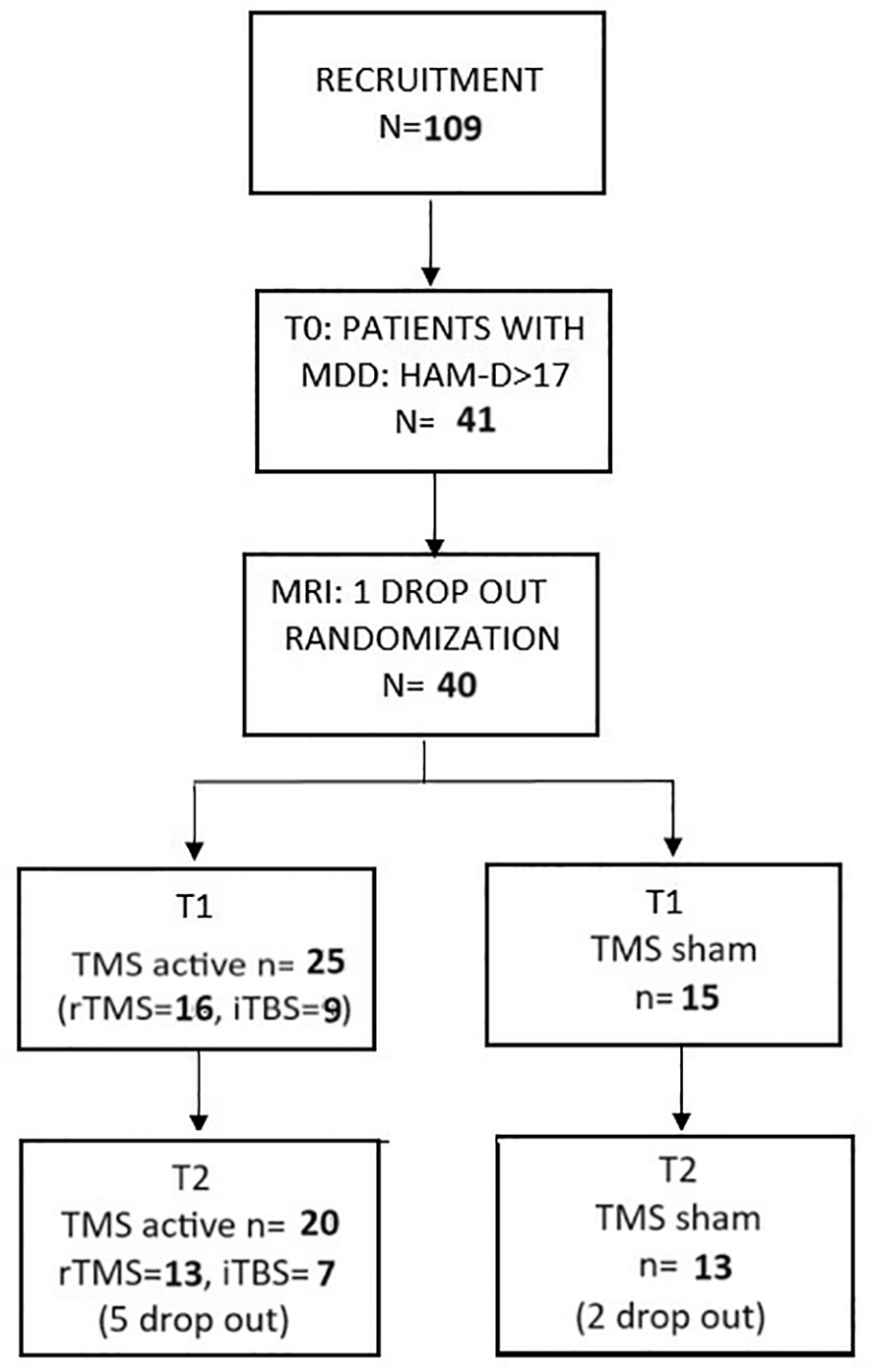
The CONSORT diagram.

During recruitment, a total of 109 patients were examined by a psychiatrist at T0. Only 41 of those patients were found to meet the inclusion criteria and proceeded to get an fMRI. One patient was excluded from the study at this point due to radiological contraindications. Finally, a total number of 40 patients were enrolled in the first phase of our study and were randomly assigned to one of two types of stimulation: A—TMS active (*n* = 25) and B—TMS sham (*n* = 15). All patients finished their stimulation protocols and underwent another psychiatric evaluation after stimulation (T1). A dropout of seven patients between T1 and T2 (3 months of follow-up) was noted, all of which were due to non-compliance, leaving a total of 33 patients to be analyzed at T2. The CONSORT diagram representing the study design can be found in **Figure 1**.

### 3.2 Descriptive statistics and general characteristics of the studied group

The mean age of the studied group (*n* = 35) was 38.4 (± 12.7) years, with an equal sex distribution and the mean years of education estimated at 15.7 ( ± 1.9). The patients reported that their mean disease duration was 14.0 years ( ± 9.9) with an average score of 27.5 ± 6.6 in MADRS at baseline (T0). The average motor threshold was 48.0% ( ± 6.5) of the device’s maximal output. With the exception of one patient, all patients had been on stabilized pharmacotherapy for at least 1 month prior to recruitment and remained on these drugs until the end of the stimulation and the study with no change in dosage: 3 patients received tricyclic antidepressants (amitriptyline), 12 patients selective norepinephrine reuptake inhibitors (SNRIs) (duloxetine: 4, venlafaxine: 8), 4 patients used bupropion, and 25 patients were prescribed medications from the selective serotonin reuptake inhibitor (SSRI) group (trazodone: 6, paroxetine: 5, sertraline: 5, fluoxetine: 2, escitalopram: 4, vortioxetine: 2). Additionally, 6 individuals incorporated pregabalin, 3 individuals lamotrigine, and 3 individuals were administered diazepam. The psychological examination using the SCID-5-PD structured interview revealed comorbid PDs in 15 patients (37.5%). In all patients, the dominant diagnosis was major depression, while personality disorders were an additional diagnosis. The following types of PDs were found in the group: borderline PD (BPD, *n* = 8), narcissistic PD (NPD, *n* = 5), and histrionic PD (HPD, *n* = 2). Additionally, there was one patient with mixed PD features, exhibiting BPD, NPD, and antisocial PD traits. There were no significant differences between the randomized groups regarding sociodemographic variables, motor threshold, severity of depressive symptoms at baseline (T0), and presence of a comorbid PD. The detailed general characteristics of the study sample including depressive symptoms severity at baseline (T0), after stimulation (T1), and after 3 months of follow-up (T2) with discernment on the active and sham groups can be found in **Table 1**.

**Table 1:** Comparison of the TMS active and sham groups and the general characteristics of the study sample.

Variable	Total (*n* = 34)	TMS active (*n* = 21)	TMS sham (*n* = 13)	*p*
**Age**	38,4 ± 12.7	39.0 ± 14.6	39.5 ± 9.2	0.941
**Sex (female)**	17 (50.0%)	6 (46.2%)	11 (52.4%)	0.743
**Education level (years of education)**	15.7 ± 1.9	15.3 ± 1.7	16.2 ± 2.1	0.150
**Disease duration (years)**	14.0 ± 9.9	12.6 ± 10.9	16.2 ± 7.9	0.118
**Motor threshold (%)**	48.0 ± 6.5	49.0 ± 7.0	46.4 ± 5.6	0.320
**MADRS T0**	27.5 ± 6.6	28.6 ± 7.1	25.8 ± 5.6	0.320
**MADRS T1**	15.4 ± 10.5	15.0 ± 10.8	15.8 ± 10.5	0.845
**MADRS T0–T1 (%)**	44.8 ± 34.1	47.9 ± 34.4	39.9 ± 34.3	0.595
**MADRS T2**	15.1 ± 9.6	13.9 ± 10.9	16.8 ± 7.5	0.429
**MADRS T0–T2 (%)**	41.0 ± 36.4	45.7 ± 41.6	34.2 ± 27.6	0.521
**Coexisting personality disorder (diagnosed personality disorder)**	15 (44.1%)	10 (47.6%)	5 (38.5%)	0.621

Data expressed as *n* (%) or mean (SD).

### 3.3 Depressive symptoms

There were no significant differences between the TMS active and TMS sham groups in terms of general characteristics, coexisting personality disorders, and MADRS scores before stimulation (T0), at the end of stimulation (T1), and after 3 months of stimulation (T2). The average MADRS score reduction from baseline after stimulation (T1) in the active TMS group was 47.9% ± 34.4% and 39.9% ± 34.3% in the sham group. The average MADRS score reduction from baseline after 3 months of follow-up (T2) was 45.7% ± 41.6% in the active TMS group and 34.2% ± 27.6% in the sham TMS group. The ANCOVA did not reveal a significant effect of group (active vs. sham TMS) on the level of reduction of depressive symptoms at the end of stimulation and after 3 months of follow-up after co-varying for the effects of potential confounding factors (**Table 2**). There was a significant independent effect of personality disorders in the ANCOVA model related to the end of stimulation (*F* = 18.8, *p* < 0.001), whereas it was not observed in the ANCOVA model related to 3 months of follow-up.

**Table 2:** Reduction of MADRS score from baseline to end of stimulation and after 3 months of follow-up after adjustment for potential confounding factors (ANCOVA).

	End of stimulation (T1)		3-month follow-up (T2)	
	*F*	*p*	*F*	*p*
Baseline MADRS score	2.572	0.122	0.011	0.917
Active TMS	1.098	0.306	0.664	0.426
Age	0.121	0.732	0.047	0.832
Education years	0.244	0.626	0.342	0.566
Sex (female)	1.316	0.263	0.273	0.608
MT %	0.396	0.535	1.165	0.296
TMS type (rTMS)	0.417	0.525	1.604	0.222
Coexisting personality disorder	18.821	<0.001	1.253	0.279
Disease duration (years)	0.533	0.473	1.851	0.191

### 3.4 Linear regression—a *post-hoc* exploratory analysis for potential confounders

**Figure 2:**
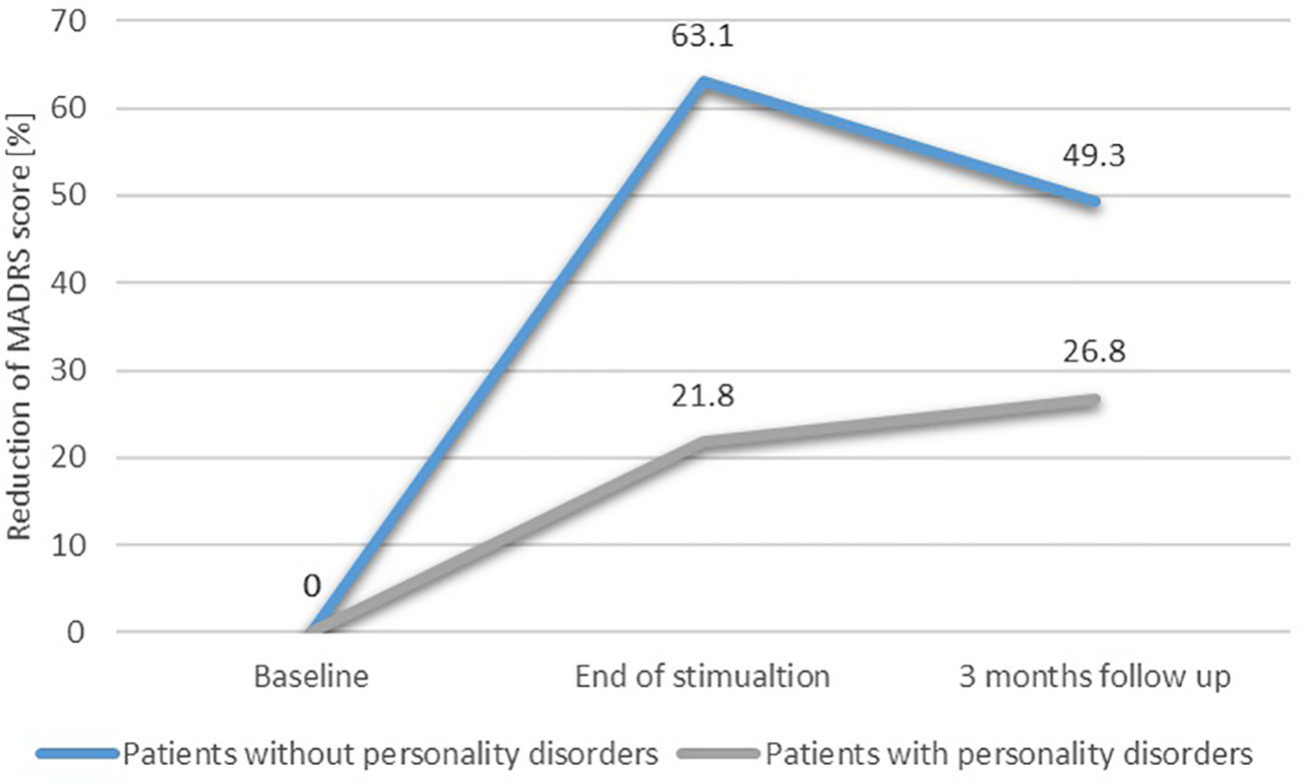
Reduction of depressive symptoms depending on the occurrence of personality disorders.

In order to find potential confounding factors of TMS efficacy, we performed a *post-hoc* exploratory analysis. Two linear stepwise regressions of bivariate variables were carried out. The first one included the reduction of MADRS scores from baseline to T1 (**Table 3**), while the second one included the reduction of MADRS scores from baseline to T2 (**Table 4**). The stepwise procedure included two blocks of independent variables: 1) TMS factors and sociodemographic variables (TMS active or sham protocol, type of stimulation: rTMS or iTBS, MT, age, sex, years of education) and 2) clinical characteristics (personality disorder coexistence, depression duration, baseline depression severity). Collinearity was assessed by calculating the variance inflation factor (VIF). Significant collinearity was defined as VIF >4. Linear regression analysis (*R* ^2^ = 0.815) revealed significant negative associations between the coexistence of PD and the reduction of depressive symptoms from T0 to T1 (Beta = −47.603; *p* < 0.001). There were no other significant factors across all models. **Figure 2** presents the reduction of the MADRS total score depending on the occurrence of personality disorders.

**Table 3:** Linear stepwise regression—dependent variable: reduction of MADRS score from baseline to the end of stimulation.

Model	Variable	Beta	*t*	*p*	VIF
TMS factors and sociodemographic variables *R*² = 0.660	Age	0.007	0.347	0.731	1.034
	Education years	−1.360	−0.343	0.734	1.032
	Sex (female)	4.968	0.354	0.726	1.040
	MT %	0.415	0.408	0.687	1.035
	iTBS	31.439	0.353	0.727	1.185
	rTMS	18.550	0.216	0.831	1.393
	Active TMS	10.040	0.715	0.481	1.533
Clinical characteristics *R*² = 0.815	Age	−0.006	−0.347	0.732	1.244
	Education years	−1.563	−0.494	0.626	1.647
	Sex (female)	13.190	1.147	0.263	1.219
	MT %	0.509	0.629	0.535	1.699
	iTBS	11.261	1.443	0.163	1.624
	rTMS	10.499	1.375	0.182	1.195
	Active TMS	11.720	1.048	0.306	1.238
	Coexisting personality disorder	−47.603	−4.338	**<0.001**	1.432
	Disease duration (years)	−0.421	−0.730	0.473	1.973
	Baseline MADRS score	−1.412	−1.604	0.122	1.143

Data expressed as *n* (%) or mean (SD). Significant differences (*p* < 0.05) were marked with bold characters.

**Table 4:** Linear stepwise regression—dependent variable: reduction of MADRS score from baseline to 3 months of follow-up after stimulation.

Model	Variable	Beta	*t*	*p*	VIF
TMS factors and sociodemographic variables *R*² = 0.640	Age	0.519	0.768	0.452	1.261
	Education years	3.289	0.613	0.547	1.005
	Sex (female)	12.596	0.807	0.429	1.393
	MT %	1.422	1.146	0.265	1.040
	iTBS	−12.766	−0.803	0.432	1.035
	rTMS	−11.955	−0.815	0.424	1.185
	Active TMS	17.036	0.972	0.343	1.533
Clinical characteristics *R*² = 0.699	Age	−0.201	−0.216	0.832	1.244
	Education years	3.180	0.585	0.566	1.647
	Sex (female)	8.628	0.522	0.608	1.219
	MT %	1.557	1.079	0.296	1.699
	iTBS	34.283	0.185	0.855	1.624
	rTMS	32.957	0.173	0.865	1.195
	Active TMS	14.254	0.815	0.426	1.321
	Coexisting personality disorder	−18.943	−1.119	0.279	1.432
	Disease duration (years)	−1.642	−1.360	0.191	1.973
	Baseline MADRS score	−0.181	−0.105	0.917	1.142

Data expressed as *n* (%) or mean (SD).

## 4 Discussion

The aim of our study was to compare the efficacy of active TMS and sham–placebo protocols in combined therapy of TRD taking into account the co-occurrence of personality disorders.

The direct analysis of the protocols’ efficacy has not yielded significant results. Hence, a *post-hoc* exploratory analysis was performed in order to establish whether personality disorders can be a potential confounding factor of active and sham TMS efficacy, which in turn indicated a strong, consistent negative influence of coexisting PDs on MADRS scores between baseline and the end of stimulation. This result stands in contradiction to previous studies on the influence of PDs on TMS treatment (26, 41–43); it is, however, in line with a battery of research pointing toward poorer outcomes in the treatment of depression for patients with PDs (53) and with the finding of Bulteau et al. (59) who concluded that the influence of personality disorders on the negative self-reference domain highlights the significance of this variable in the prognosis of MDD, irrespective of the biological treatment modality employed.

In the only well-powered study on PDs in high-intensity TMS treatment regarding left DLPFC available, Ward et al. (55) concluded that comorbid BPD did not influence the efficacy of TMS. This study, however, apart from the undoubted advantage of a large study group, had a number of limitations, including the lack of a uniform protocol, different stimulation durations, no placebo control, and the fact that the BPD diagnosis was not based on a clinical examination by a psychologist but resulted from a retrospective self-report questionnaire. Our results are supported by the RCT study design, as well as the PD diagnosis, which was based on clinically structured SCID-5-PD interviews that were conducted before stimulation. Therefore, we believe that in the absence of alternative studies, despite the small size of the groups, these results provide new information on the impact of PDs on the efficacy of TMS in TRD treatment.

In a meta-analysis conducted by Newton-Howes et al. (54), the authors concluded that a coexisting diagnosis of a personality disorder had a negative impact on the outcome of depression treatment across treatment modalities. Interestingly, the aforementioned meta-analysis did not discriminate between types of intervention and, therefore, pooled data from prospective case series and as well as randomized controlled trials addressing the efficacy of various methods, such as electroconvulsive therapy, counseling, cognitive–behavioral and psychodynamic treatments, and various pharmacological interventions (e.g., selective serotonin reuptake inhibitors, tricyclic antidepressants, and monoamine oxidase inhibitors). The only common factor was that the treatments, similar to the treatments examined in our study, exclusively targeted depressive symptoms disregarding the underlying PD in terms of intervention.

No difference in response between sham and active stimulation is in contradiction with previous RCTs, which mostly point to a higher efficacy of both active rTMS and active iTBS protocols compared to a sham–placebo group (1, 7).

Addressing the question about the possible reasons for the lack of difference in antidepressant effects of active and sham stimulation, we should be aware of the limitations of our study (discussed later), namely, the relatively low sample size and methodological factors including combining into one group participants receiving active rTMS and active iTBS stimulation and, lastly, the presence of confounding factors such as personality disorders.

At this point, apart from the previously mentioned impact of PDs on the treatment of depression, the study’s demographic regarding the prevalence and distribution of personality disorders must be considered. Based on a recent systematic review and meta-analysis, it is estimated that the worldwide pooled prevalence of PDs is 7.8% (95% CI 6.1–9.5) of the population, with the most common being cluster C with 5.0%, followed by cluster A with 3.8%, and the least common globally, cluster B with 2.8% (67). In our study, we found 15 patients (37.5%) to have a co-occurring PD, all of which fell under cluster B personality disorders (BPD in *n* = 8, NPD in *n* = 5, HPD in *n* = 2, and mixed BPD, NPD, and antisocial PD traits in *n* = 1). Our study sample therefore seems to be disproportionately skewed toward personality disorder patients with a total overrepresentation of B cluster patients (100%). According to research, comorbid PDs are one of the factors associated with TRD (68), and B cluster personality disorders are associated with poorer depression outcomes. In a study by George et al. (63), the results indicated that a cluster B PD diagnosis exhibited the most significant association with a worse treatment prognosis for MDD after a 6-month follow-up. Therefore, this disproportion could explain the lack of efficacy of either treatment modality employed in this study as well as no significant effect of stimulation after the follow-up period.

The question of why these patients, who exhibit depressive symptoms but in fact have an underlying cluster BPD, have reported to our study in such large numbers (i.e., larger than expected and almost larger than “only” TRD patients without a PD) remains. A selection bias, specifically self-selection bias, partially explains these results, where the patients who volunteered for the study may have been more likely to display cluster B personality traits. From the patient’s perspective, the study may have been considered novel and somewhat higher risk than treatment as usual. However, since we did not specifically recruit patients with PDs but with TRD, the recruitment strategies were diversified as were the clinicians, who assessed for inclusion criteria, and a biased selection at recruitment seems unlikely. A more likely explanation includes a possible bias in the targeted sample related to the inclusion criteria, more specifically, the required TRD diagnosis. Despite a well-established link between MDD, TRD, and PDs, the only conclusion remains that they are indeed related and that a comorbid PD will have a negative effect on MDD treatment prognosis. There is little mention of whether any differences in clinical presentation or patient treatment choices can be observed, and the debate on the validity of TRD diagnoses and misdiagnoses is a long-standing one (69). Furthermore, according to some clinicians, the resistance of depression to pharmacological treatment may be related to the psychogenic etiopathology (personality-related) of depressive symptoms in a certain group of patients (70).

In a similar discussion, Sedlinska et al. (71) suggest the concept of “male depression” as a possible link between depression and cluster B personality traits. Male or masculine depression (MDS) is a term that was proposed in research addressing the gender gap in diagnosis and the clinical picture of MDD between men and women. The term describes a cluster of depressive symptoms that includes traditional MDD symptoms along with additional externalizing symptoms that are typically more often exhibited by depressed men (i.e., anger, irritability, impulsiveness, substance abuse, and risk-taking behaviors) but are not limited to the male gender (72). Sedlinska et al. (71) in their study significantly associated pronounced cluster B personality with high MDS scores as opposed to patients with low MDS scores. The authors hypothesize that MDS could be understood as an exacerbation of previous cluster B personality traits (based on the phenotypical resemblance of the most prominent traits of both diagnoses), i.e., cluster B traits posing a premorbid risk factor for developing MDS (which is effectively depression with externalizing traits). In that context, we could have recruited more “male depression” cluster B PD patients as TRD patients due to several reasons. One of them is that they possibly better fit the TRD and symptom intensity requirements than no PD MDD patients, who are more likely to have fewer symptoms and have benefitted from previous treatments before. Another is the fact that more externalizing symptoms may contribute to more frequent contact with the psychiatric environment, i.e., a higher accessibility to information regarding the study. Additionally, patients with more externalizing symptoms may be more prone to take an active approach to seek novel and experimental treatments than MDD patients with more passive internalizing symptoms. As we did not record any additional data on the patients’ specific symptoms, confirming this hypothesis would require additional studies considering the specific clinical picture of the diagnosed TRD. In conclusion, PDs are either specified as an exclusion criteria or they must be specifically controlled for in order to evaluate treatment outcomes in MDD and TRD populations to avoid additional confounding variables.

A potential psychodynamic explanation of our results may lie in the presence of a defense mechanism of idealization and devaluation characterizing both patients with BPD and NPD, which would prompt them to an initial phase of short-term fascination with the stimulation and then lead to a drastic discouragement, which in turn could inhibit psychological and neurobiological processes allowing for a reduction of depressive symptoms through stimulation (73, 74). In addition, patients with BPD who are particularly susceptible to rejection may become triggered by the end of stimulation due to an anticipated perspective of the end of the relationship with the TMS staff and study environment. This may similarly evoke negative emotions exacerbating the severity of depressive symptoms at T2. The occurrence of depressive symptoms among patients with NPD most often concerns the population of the so-called “thin skin” NPD patients, who, unlike the “thick skin” patients, are prone to experiencing narcissistic wounds in response to external factors, leading to an imbalance in an already initially unstable self-esteem often associated with the presence of depressive symptoms as consequences of the personality structure (50). This often happens as a result of the deepening of the disproportion between the grandiose self’s own quantitative expectations and reality. In this sense, TMS by not “magically” reducing the nature of narcissistic grandiose self and not directly affecting the reality of the patient’s life may be experienced by the patient as disappointing and, paradoxically, lead to an aggravation in depressive symptoms, which was indeed subjectively reported by some of our patients. From a psychodynamic perspective, the long-term ineffectiveness of TMS among patients with PDs may result from the negative impact of maladaptive defense mechanisms resulting from unresolved internal conflicts, identity, and object relationship disorders that persist despite stimulation and cause secondary depressive symptoms in the follow-up (73, 74).

Unfortunately, none of the 3-month follow-up results were statistically significant, which we largely attribute to the small sample size, and therefore cannot be treated as a base to draw further conclusions. A comparison of the patients’ scores and the percentage of symptom reduction, however, suggests a logical picture indicating that patients without PDs achieve a greater degree of long-lasting improvement for at least 3 months after the end of stimulation, while patients with comorbid PDs experience a much smaller improvement and are unable to maintain it for the follow-up period. This observation may refer to the phenomenon of neuroplasticity, which is considered the basis for a maintained TMS efficacy, independent of the placebo effect, for at least 3 months after the end of stimulation (75). The confirmation of this hypothesis would require additional studies comparing the effects of stimulation in patients with and without PDs, in particular regarding the concentration of brain-derived neurotrophic factor (BDNF) and brain activity before, after, and at 3 months of follow-up after stimulation (75). This hypothesis may also be supported by neuroscientific data concerning BPD patients, who exhibit a dysfunction of the fronto-limbic circuitry and the default mode network (47, 48), while patients with MDD without PDs are characterized by an increased effective connectivity within the central executive network and a decreased connectivity in regions of the central executive network to the default mode network (76). The difference in brain connectivity may also serve as a theoretical explanation for the varying antidepressant responses to TMS among patients with and without comorbid BPD.

In our study, no significant difference in symptom reduction was observed between the active TMS group and the placebo group, aligning with the findings from Lefaucheur et al. and Cirillo et al., which noted that short-term clinical outcomes between active TMS or TBS and sham treatments can be comparable (77, 78). However, electrophysiological indicators such as motor-evoked potentials (MEPs) or brain oscillations are often more sensitive to TMS effects (79, 80). High-frequency rTMS typically increases MEP amplitudes, indicating enhanced cortical excitability, while iTBS leads to more rapid and sustained excitability changes, according to Wilson et al. and Fitzgerald et al. (81, 82). High-frequency rTMS is associated with increased power in higher-frequency bands, such as beta and gamma, indicating enhanced cortical excitability and synchronization of neural circuits involved in mood regulation (83). These electrophysiological changes, particularly in brain oscillations, offer valuable insights into the neurobiological effects of TMS, even when clinical improvements are not immediately apparent (83). Additionally, rTMS and iTBS used in the present study differ in their impact on corticospinal excitability and MEPs (79). High-frequency rTMS results in a gradual increase in MEP amplitude, reflecting enhanced cortical excitability (81). In contrast, iTBS produces more rapid and sustained changes in excitability, suggesting distinct neuroplasticity mechanisms and efficacy (79). The incorporation of electrophysiological measures in future TMS studies could help detect subtle neurophysiological changes that precede clinical improvement, providing a more comprehensive understanding of its therapeutic potential (84).

## 5 Limitations

The fact that our study only included a small number of study groups significantly limits the results’ capacity to be generalized. It also affects the conclusions and power of statistical analysis, particularly when it comes to the follow-up time. Due to the limited sample size, we combined the rTMS and iTBS active groups into one active TMS group, which is an important limitation. Another limitation is the retrospective analysis of the outcome, which would benefit from gathering patient motivations for participating in the study as well as addressing other variables that could inform the clinical diagnosis of the TRD (e.g., specific experienced symptoms, other than the used scales). Due to the size of the groups, we did not analyze the effect of the drugs and drug combinations used although it should be noted that all participants were on medication doses that had been stabilized for at least 1 month prior to recruitment. The use of limited clinical data related to psychological aspects and other important elements that could more fully explain the absence of response to TMS constituted another constraint. We also observed overrepresentation of PDs in our sample which could have an impact on our results.

## 6 Conclusions

Comorbid personality disorders have a significant negative impact on the efficacy of TMS stimulation in combined therapy of TRD regardless of active or placebo conditions. After stimulation, patients with co-occurring personality disorders exhibit increased depressed symptoms at the end of stimulation and a generally lower symptom reduction as a result of TMS. Based on our findings, a key finding is that TMS for depression cannot be regarded as a treatment that is independent of co-occurring personality disorders. This emphasizes the significance of careful recruitment processes to guarantee that patients who are recommended for TMS treatment will be able to gain both immediate and long-term benefits from the intervention. Considering the limitations of our study, more extensive research is required, including assessments of concomitant PDs at the time of enrollment as well as analyses of the association of specific antidepressants with stimulation. Future research on the combination of psychotherapy and TMS for patients with comorbid PDs and TRD would be relevant if the outcomes of this study could be replicated in other research.

## Data Availability

The raw data supporting the conclusions of this article will be made available by the authors, without undue reservation.
